# High Sensitive Cardiac Troponin-I (Hs-cTnI) Levels in Asymptomatic Hemodialysis Patients †

**DOI:** 10.3390/jcm14155470

**Published:** 2025-08-04

**Authors:** Ofir Rabi, Linda Shavit, Ranel Loutati, Louay Taha, Mohammad Karmi, Akiva Brin, Dana Deeb, Nir Levi, Noam Fink, Pierre Sabouret, Mohammed Manassra, Abed Qadan, Motaz Amro, Michael Glikson, Elad Asher

**Affiliations:** 1 Jesselson Integrated Heart Center, Shaare Zedek Medical Center, Eisenberg R&D Authority and Faculty of Medicine, The Hebrew University of Jerusalem, Jerusalem 9112102, Israel; ranellout@gmail.com (R.L.); louayt@szmc.org.il (L.T.); mkarmi@szmc.org.il (M.K.); akivabr@szmc.org.il (A.B.); danad@szmc.org.il (D.D.); mohammedma@szmc.org.il (M.M.); qadana@szmc.org.il (A.Q.); motaza@szmc.org.il (M.A.); mglikson@szmc.org.il (M.G.); easher@szmc.org.il (E.A.); 2 Nephrology Unit, Shaare Zedek Medical Center, Faculty of Medicine, The Hebrew University of Jerusalem, Jerusalem 9112102, Israel; lshavit@szmc.org.il; 3 Management Department, Assuta Medical Centers, Faculty of Medicine, The Hebrew University of Jerusalem, Jerusalem 9190500, Israel; noamfink@bezeqint.net; 4 Pitié Salpêtrière, Heart Institute, Cardiology Department, Action Group, Sorbonne University, 47-83 bd de l’Hopital 75013 Paris, France

**Keywords:** high-sensitivity cardiac troponin I (hs-cTnI), hemodialysis, acute myocardial infarction (AMI), biomarkers

## Abstract

**Background**: High-sensitivity cardiac troponin (hs-cTn) is useful for detecting acute myocardial infarction, but chronic hemodialysis patients often have elevated baseline levels that exceed the upper reference limit (URL). This study aimed to determine whether hs-cTnI levels in asymptomatic hemodialysis patients exceed the URL established for the general population, evaluate the impact of high-flux hemodialysis on hs-cTnI concentrations, and examine associations between hs-cTnI levels and subsequent hospitalization or mortality. **Methods**: A prospective, single-center cohort study was conducted at a tertiary care center from August 2023 to July 2024. Blood samples for hs-cTnI were collected from asymptomatic hemodialysis patients aged ≥ 40 years, measured before and after dialysis within one month. Patients were followed for up to 12 months. **Results**: Fifty-six patients were enrolled. The mean hs-cTnI levels were 28.4 ng/L pre-dialysis and 27.9 ng/L post-dialysis, with ranges of <6–223 ng/L and <6–187 ng/L, respectively. The mean hs-cTnI delta between pre- and post-dialysis was −0.5 ng/L, with 52% showing a negative delta, 30% no change, and 18% a positive delta. No association was found between baseline hs-cTnI levels and mortality or hospitalization during follow-up. **Conclusions**: Most asymptomatic hemodialysis patients had hs-cTnI levels in the “gray zone”, thus neither confirming nor excluding acute myocardial infarction. Dialysis did not significantly affect hs-cTnI levels, and elevated baseline hs-cTnI was not linked to increased mortality or hospitalization over 12 months.

## 1. Introduction

Acute myocardial infarction (AMI) is defined according to the universal criteria as a combination of clinical symptoms; characteristic ECG changes; and changes in cardiac enzyme levels, preferably troponin, above the 99th percentile of the upper reference limit (URL) [1]. In recent years, the increasing use of high-sensitive cardiac troponin (hs-cTn) has significantly improved the early diagnosis of AMI [2]. However, the reference values for troponin tests are based on studies conducted on healthy populations and may not adequately represent patients with chronic diseases, particularly those with chronic kidney disease (CKD).

Evidence suggests that basal troponin levels in patients with CKD are higher than those in the general population and may even exceed the 99th percentile [3]. Moreover, studies have demonstrated that most patients undergoing chronic hemodialysis exhibit troponin levels above the URL of the test [4]. Despite this, adjusted reference values for these patients are not currently available, making the diagnosis of AMI in this population particularly challenging. When such patients present symptoms suggestive of a cardiac event, they are at risk of being misdiagnosed with AMI or experiencing delays or missed diagnoses due to the erroneous assumption that elevated troponin levels are solely attributable to chronic dialysis [5]. There is also evidence suggesting that elevated troponin levels are not necessarily indicative of an acute condition or a specific clinical context. For example, certain patients—such as those with chronic cardiovascular disease—may exhibit chronically elevated troponin levels, which are often subclinical and do not reflect ongoing myocardial injury [6].

Several studies have aimed to establish appropriate troponin reference values for dialysis patients [4,7,8,9,10], most of which have focused on “regular” troponin tests rather than high-sensitivity troponin tests [8]. Additionally, the evidence regarding changes in troponin levels following hemodialysis is conflicting [3]. While some studies report a decrease in troponin levels post-hemodialysis [11], others observe no change [12], and some even report an increase [13]. Furthermore, elevated baseline levels of high-sensitivity cardiac troponin T (hs-cTnT) and high-sensitivity cardiac troponin I (hs-cTnI) have been associated with worse prognoses in asymptomatic dialysis patients [9,14].

Therefore, the aim of the current study was to assess whether hs-cTnI in asymptomatic hemodialysis patients exceed the established URL values in the general population, evaluate the impact of hemodialysis on hs-cTnI levels, and assess the association between hs-cTnI levels and hospitalization and mortality rates up to a 12-month follow-up period.

## 2. Methods

### 2.1. Study Population

A prospective single-center observational cohort study was performed in a tertiary care center between August 2023 and July 2024.

Inclusion criteria: Asymptomatic patients aged ≥40 years with end-stage renal disease (ESRD), defined by the requirement for chronic maintenance hemodialysis for at least 90 days, were enrolled.

Exclusion criteria: Patients diagnosed with AMI, clinical heart failure (newly diagnosed heart failure, with either preserved or reduced ejection fraction, or a hospitalization for acute decompensation of pre-existing heart failure), pulmonary embolism, or had undergone coronary and/or valvular interventions 6 months prior to enrolment were excluded. Additionally, patients who had undergone major surgery or experienced trauma within 4 weeks prior to enrolment were not eligible.

### 2.2. Clinical Data and Study Outcome

As this is an observational study, all clinical management decisions were made exclusively by the treating nephrology team. Data were collected anonymously by the local coordinator and prospectively submitted into an electronic case-report form (eCRF). Variables systematically recorded included demographic data, comorbid conditions, the most recent echocardiographic findings available in the medical records prior to the time of sample collection, suspected cause of renal failure, type of dialyzer, dry weight, intradialytic weight gain, duration of hemodialysis, dialysis vintage, dialysis efficacy (Kt/V), and laboratory results. All reported variables were sourced from the patients’ medical records in the hospital′s dialysis unit.

Hs-cTnI levels were measured in a central laboratory using the ARCHITECT STAT hs-cTnI immunoassay, with a 99th percentile reference value of 17 ng/L for females and 35 ng/L for males. Diagnosis or exclusion of cardiac events followed the latest European Society of Cardiology (ESC) guidelines, using the “rule-out” (hs-cTnI < 6 ng/L) and “rule-in” (hs-cTnI ≥ 64 ng/L) thresholds [15,16].

Hs-cTnI samples were collected immediately before the initiation of dialysis and again immediately upon its completion, as part of routine monthly testing in the hemodialysis unit. Two paired sampling events—pre- and post-dialysis—were performed for each patient, spaced one month apart. This design aimed to ensure the consistency and reliability of hs-cTnI measurements and the calculated delta between them, thereby minimizing the likelihood that the observed values resulted from random variation. The hs-cTnI delta was then calculated, with a positive delta defined as an increase in hs-cTnI levels following dialysis, and vice versa.

### 2.3. Patients Follow-Up

The mean follow-up duration was 8.46 ± 3.29 months. Patients were monitored for mortality, kidney transplants, and hospitalizations for any reason.

### 2.4. Statement of Ethics

This study was performed in accordance with the Declaration of Helsinki. This study was approved by the institutional review board at the Shaare Zedek Medical Center (approval ID: 0002-23-SZMC). The study was not registered as a clinical trial because this is a prospective observational cohort study. As this was a prospective observational cohort study utilizing routine monthly pre- and post-dialysis blood samples collected for clinical purposes, no additional blood draws were performed. Patients were informed of the additional use of their routine samples, and verbally informed consent was obtained and documented in the research protocol folder. This consent procedure was reviewed and approved by the institutional review board, approval number 0002-23-SZMC, with the decision date being 9 May 2023. The verbal consent was documented in the research protocol folder.

### 2.5. Statistical Analysis

Continuous variables were expressed as mean ± standard deviation if normally distributed, or median with interquartile range if skewed. Categorical variables were presented as frequency (%). Continuous data were compared with Student’s *t*-test, and categorical data were compared with the use of the Chi-square test or Fisher’s exact test where appropriate. Differences between the three delta hs-cTnI groups were analyzed using one-way ANOVA for continuous variables that were normally distributed, while the Kruskal–Wallis test was used to compare continuous variables that did not adhere to a normal distribution.

For survival analysis, patients were censored only in the case of death during follow-up. Univariate Cox proportional hazards regression was used to evaluate mortality among patients with pre- or post-dialysis hs-cTnI levels above the median compared to those with hs-cTnI levels below the median. Similarly, univariate logistic regression was employed to assess the risk of hospitalization within 90 days for patients with pre- or post-dialysis hs-cTnI levels above the median compared to those below the median. All analyses were performed using R software version 4.3.3 (R Foundation for Statistical Computing). An association was considered statistically significant for a two-sided *p*-value of less than 0.05.

## 3. Results

During the study period, 56 hemodialysis patients were enrolled. Patients′ baseline characteristics are presented in Table 1. Mean age was 71.8 (±12.87), and 35 (62.5%) patients were male. Hypertension was present in 51 (91%) patients, while 35 (62.5%) had diabetes mellitus (DM). Additionally, 25 (45%) patients had a history of coronary artery disease, and 27 (48%) had chronic heart failure, the majority of whom had preserved left ventricular ejection fraction (LVEF) (70.4%).

### 3.1. Dialysis Characteristics

Dialysis characteristics are presented in Table 2. In total, 52 (93.0%) patients underwent high-flux dialysis, while 54 (96.4%) patients attended three hemodialysis sessions per week. Blood flow during hemodialysis was 300 mL/min in 53 (94.7%) patients. The mean dialysis duration was 3.65 ± 0.41 hours, and the mean Kt/V was 1.38 ± 0.25.

### 3.2. Hs-cTnI Levels

**Figure 1:**
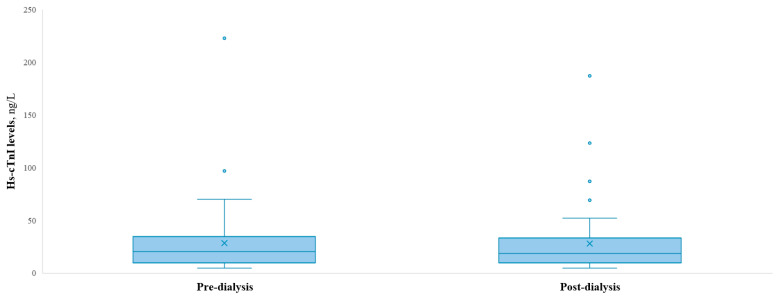
Hs-cTnI levels pre- vs. post-dialysis.

The mean hs-cTnI levels pre- and post-dialysis were 28.4 (±32) ng/L and 27.9 (±30) ng/L, respectively, as shown in Figure 1. Hs-cTnI level ranges for pre- and post-dialysis were <6–223 ng/L and <6–187 ng/L, respectively. In total, 4 patients (7.14%) had a pre-dialysis hs-TnI levels below the detectable threshold (<6 ng/L), 3 (5.35%) had levels above the “rule-in” MI threshold (>64 ng/L), and the remaining 49 (87.5%) patients had levels within the “gray zone” (>6 and <64 ng/L). Hs-cTnI mean levels after one month pre- and post-dialysis were 25.67 (±24) ng/dL and 26.37 (±25) ng/dL, respectively.

**Figure 2:**
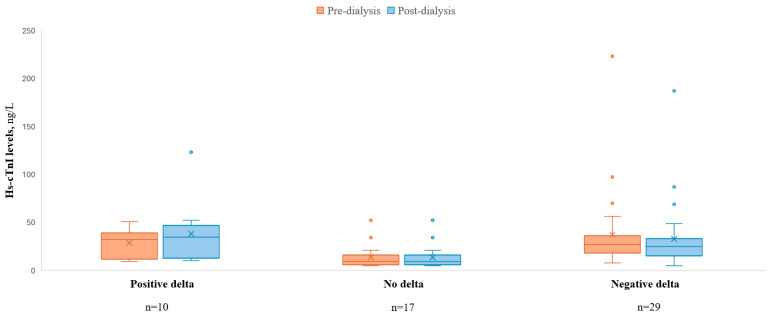
Hs-cTnI levels pre- vs. post-dialysis by delta troponin.

The mean hs-cTnI delta between pre- vs. post-dialysis was −0.5 (±11.63) ng/L, with a range of (−12)–36 ng/L (minus twelve to thirty-six). Twenty-nine (52%) patients had a negative delta, while seventeen (30%) had no delta and ten (18%) had positive delta (i.e., increase hs-cTnI) post-dialysis (Figure 2). In the second set of measurements collected one month later, the mean hs-cTnI delta was 0.7 ± 4 ng/L.

### 3.3. Hs-cTnI and “Gray Zone” Levels

Among the 10 patients with a positive delta, all were in the “gray zone” prior to dialysis (6 < hs-cTnI < 64 ng/L). One patient exhibited an increase in hs-cTnI that shifted them into the “rule-in MI” category (from 51 to 123 ng/L). None of the 29 patients with a negative delta shifted from their original category after dialysis.

The median pre-dialysis hs-cTnI level was 20.5 ng/L. Patients with hs-cTnI levels above the median were older (74.9 ± 12.9 vs. 68.7 ± 12.3, *p* = 0.034) and had lower creatinine levels both pre-dialysis (6.52 vs. 7.87 mg/dL, *p* = 0.019) and post-dialysis (2.30 vs. 2.88 mg/dL, *p* = 0.011). They also had a higher prevalence of hypertension as the cause of ESRD (32.1% vs. 0.0%, *p* = 0.013). Additionally, these patients experienced less intra-dialytic weight gain (1.3 vs. 1.47 kg, *p* = 0.03) and had a lower dialysis target weight loss (2.43 vs. 2.83 kg, *p* = 0.047), as summarized in Table 3.

### 3.4. Outcomes

During the follow-up period, 10 (17.9%) patients died, and 23 (41%) were hospitalized, including 5 (9%) due to cardiac complaints such as congestive heart-failure exacerbation and arrhythmias. It is important to note that none of the patients underwent invasive procedures, including percutaneous coronary intervention (PCI), during the study period. Additionally, two (3.6%) patients received a kidney transplant. No significant association was found between elevated baseline hs-cTnI levels and mortality or hospitalization rates.

## 4. Discussion

Our study provides important insights into the levels of hs-cTnI in asymptomatic hemodialysis patients, their variations in response to hemodialysis, and their association with clinical outcomes. Our findings emphasize the complexities of using hs-cTnI as a biomarker for diagnosing AMI in this specific patient population, given the unique metabolic and physiological factors associated with hemodialysis. The main findings in our study were (1) mean pre- and post-dialysis hs-cTnI levels were 28.4 ng/L and 27.9 ng/L, respectively; (2) the majority of patients (87.5%) had hs-cTnI levels within the “gray zone”; (3) most patients (52%) exhibited a negative delta in hs-cTnI levels post-dialysis, while only a minority (18%) had an increase in hs-cTnI levels; and (4) no association was found between elevated hs-cTnI levels at baseline and mortality or hospitalizations during the follow-up period.

### 4.1. Baseline hs-TnI Level in Hemodialysis Patients

The mean pre-dialysis hs-cTnI level of 20.5 ng/L aligns with previous findings showing elevated troponin levels in hemodialysis patients compared with the general population [7,8,9]. This elevation is likely influenced by factors such as chronic inflammation, left ventricular hypertrophy, and underlying vascular changes common in hemodialysis patients [17,18,19]. However, the lack of an established adjusted hs-cTnI reference value complicates its diagnostic utility [20].

Interestingly, only a small proportion of hemodialysis patients (5.3%) had baseline hs-cTnI levels within the “rule-in” range for AMI or within the normal range (7.14%). Most patients′ levels were in the “gray zone”, between the “rule-out” and “rule-in” thresholds. Furthermore, the average hs-cTnI levels and delta after one month were nearly identical in the same patients, reinforcing the validity of the initial troponin measurements, as the results were consistent even after one month. Although transient fluctuations in hs-cTnI levels may occur over the hours or days following dialysis, the present study was not designed to capture such short-term variability. Nevertheless, the minimal immediate post-dialysis changes observed, combined with the consistency of values at one-month follow-up, suggest that no clinically significant variation occurs during the intervening period.

### 4.2. Hemodialysis Effect on the hs-TnI Levels

Our observations suggest that dialysis itself has variable effects on hs-cTnI, with some patients showing stable levels while others exhibit changes. Despite the troponin I molecule’s small size (26 kD), which would typically allow for easy filtration, the levels remained stable. Kolland et al. proposed that factors such as cTnI’s high electrical charge, its binding to blood proteins, or absorption by the dialysis membrane might hinder its filtration [20].

Most patients showed a negative delta, indicating that dialysis slightly reduced hs-cTnI levels. Additionally, the majority of patients did not shift from the rule-in, rule-out, or “gray zone”. In patients whose hs-cTnI levels remained unchanged or increased, this could be attributed to myocardial stunning (recurrent segmental ischemic injury), which may result from intradialytic hypotension [21]. Furthermore, since most of the patients had DM, they may have experienced silent ischemic events during dialysis. However, no significant difference in blood pressure reduction was observed in these patients based on the delta mean arterial pressure.

A review of the literature identified three studies comparable to ours that investigated hs-cTnI levels in asymptomatic hemodialysis patients. Two of these studies, similar in methodology, measured hs-cTnI levels immediately before and after dialysis [3,4]. Wongcharoen et al. reported findings consistent with ours, both in absolute hs-cTnI concentrations and in the minimal, non-significant delta observed between pre- and post-dialysis measurements [4]. In contrast, Tarapan et al. observed higher pre-dialysis hs-cTnI levels—though still within the diagnostic “gray zone”—and a more pronounced delta. These differences may reflect heterogeneity in patient characteristics, though definitive conclusions cannot be drawn [3]. The third study, conducted by Snaedal, et al., assessed hs-cTnI only before dialysis and reported levels comparable to our own pre-dialysis values [9].

Notably, only the study by Wongcharoen et al. employed an additional set of pre- and post-dialysis blood samples, as in our protocol [4]. However, their aim was to assess whether hs-cTnI levels varied between short and long interdialytic intervals, whereas our repeated sampling was designed to evaluate measurement reproducibility and reliability. Their results revealed no significant difference in hs-cTnI levels between the two interdialytic intervals.

The lack of a uniform directional change in hs-cTnI levels following dialysis, as supported by both our findings and the heterogeneous results reported in prior studies, underscores the complexity of the physiological mechanisms involved and highlights the need for further investigation.

### 4.3. Clinical Associations

Our study also examined associations between hs-cTnI levels and other clinical and demographic variables. Elevated hs-cTnI levels were associated with older age, higher rates of pulmonary hypertension, and lower creatinine levels. The relationship between older age and high hs-cTnI levels is consistent with previous studies [22,23,24]. Lan et al. noted that age and cardiovascular comorbidities contribute to elevated baseline hs-cTnI levels. Additionally, we found that lower creatinine levels were associated with higher hs-cTnI. This might be due to the fact that creatinine levels in dialysis are linked to the patient′s muscle mass and their dietary nutrition [25]. Moreover, in older patients, muscle mass tends to be lower, which may help explain the findings, particularly since the patients in this group were older.

While these associations are noteworthy, they underscore the multifactorial nature of hs-cTnI elevations in hemodialysis patients, which may reflect not only age or nutritional status but also underlying subclinical cardiovascular dysfunction. For example, in dialysis patients without known coronary artery disease, elevated troponin levels have been associated with increased carotid intima–media thickness, suggesting that such elevations are not necessarily benign but may indicate a greater burden of cardiovascular morbidity [6].

It is noteworthy that the patient group in this study was generally homogeneous with respect to baseline characteristics and dialysis-related factors. Despite this, no significant association was found between elevated hs-cTnI levels and mortality or hospitalization rates during the follow-up period. This suggests that while hs-cTnI may reflect subclinical cardiac changes or the systemic effects of ESRD and hemodialysis, it may not directly predict adverse clinical outcomes in asymptomatic hemodialysis patients. This is consistent with previous evidence suggesting that hs-cTnT, rather than hs-cTnI, is more strongly correlated with worse prognosis in this population [9]. However, the lack of association with clinical outcomes may be attributed to the relatively short follow-up period, as longer-term studies have demonstrated a negative prognostic association between high troponin levels and clinical outcomes [26].

### 4.4. Diagnostic Challenges

One of the critical challenges highlighted by this study is the difficulty in interpreting elevated hs-cTnI levels in hemodialysis patients, particularly because these patients often have chronically elevated levels that may surpass the 99th percentile due to causes unrelated to AMI [27,28]. This has implications for clinical practice, as misinterpretation of these levels could lead to unnecessary testing, delayed diagnosis of true AMI, or overtreatment [5,7,8].

To date, there are no specific recommendations to measure baseline troponin in dialysis patients [29]. While the current European Society of Cardiology (ESC) and American Heart Association (AHA) guidelines do not recommend specific thresholds for hs-cTnI in hemodialysis patients to rule out acute MI, the increasing prevalence of advanced age and hemodialysis use may warrant the development of updated guidelines with specific “rule-in” and “rule-out” thresholds for acute MI in this patient population [20]. However, our findings suggest that hs-cTnI thresholds should not solely rely on general population reference ranges. Instead, tailored diagnostic criteria that account for the chronic physiological changes associated with ESRD and hemodialysis should be considered. This approach could improve diagnostic accuracy and ensure appropriate management of patients presenting with cardiac concerns. Another potential approach could involve placing greater emphasis on the delta hs-cTnI—the change in levels between two consecutive measurements—rather than relying solely on adjusted hs-cTnI values for diagnosing or excluding AMI in hemodialysis patients. In this study, the range of hs-cTnI values was relatively broad, likely reflecting the chronic elevation of this biomarker due to non-ischemic factors associated hemodialysis. Conversely, the delta hs-cTnI demonstrated a narrower range in these patients, making significant changes between sequential tests more indicative of an acute ischemic event. By focusing on dynamic changes in hs-cTnI levels, clinicians may improve diagnostic accuracy and reduce the risk of misclassification related to the overlapping baseline elevations observed in this population.

Lastly, our study found that most asymptomatic hemodialysis patients had hs-cTnI levels within the “gray zone”. Lim et al. showed that hemodialysis patients with ACS symptoms had hs-cTnI levels in the “rule-in MI” range (median 931 ng/dL, IQR 153-10,000 ng/dL) [5]. Therefore, patients with hs-cTnI levels in the “rule-in MI” range should be carefully evaluated for a cardiac event. Levels in the “gray zone” typically reflect the patient’s baseline but do not exclude the possibility of an AMI.

### 4.5. Study Limitations

Our study has several limitations: First, a relatively small sample size (*n* = 56) may limit the statistical power to detect associations, particularly regarding hospitalization and mortality outcomes. Second, the observational design cannot establish causality, and the generalizability of the findings to other populations or dialysis modalities remains uncertain. Third, we evaluated only hs-cTnI and not hs-cTnT. Finally, we did not follow each patient during dialysis for hemodynamic changes or symptoms and thus cannot exclude myocardial stunning during dialysis. However, no major events or symptoms were recorded in the charts during dialysis. Despite these limitations, the study was prospective and examined only asymptomatic patients, excluding those with recent AMI, heart failure, and other recent cardiac events, minimizing confounding factors. Moreover, the study’s systematic follow-up and detailed analysis of hs-cTnI changes pre- and post-dialysis provide valuable insights into the complexities of this biomarker′s role in hemodialysis patients. Additionally, by collecting an additional set of samples from the same patients, we found that the results were nearly identical after a month.

## 5. Conclusions

In summary, this study highlights that hs-cTnI levels are only mildly elevated in most asymptomatic hemodialysis patients, and they typically remain stable before and after dialysis. These results stress the importance of interpreting hs-cTnI levels within the appropriate clinical context to prevent misdiagnosis or overlooking genuine cardiac events. Additionally, our findings underscore the challenges of managing cardiovascular risk in this at-risk group and suggest the need for personalized diagnostic strategies and further longitudinal studies and guidelines recommendations.

## Figures and Tables

**Table 1:** Patients’ baseline characteristics.

	Total
	N = 56
**Age** (SD), years	71.8 (±12.9)
**Male** (%)	35 (62.5%)
**BMI** (SD)	27.7 (±5.27)
**HTN** (%)	51 (91.1%)
**DLP** (%)	47 (83.9%)
**DM** (%)	35 (62.5%)
**Smoker** ^†^ (%) (*n* = 54)	13 (23.2%)
**Prior CAD** (%)	25 (44.6%)
**Prior PCI** (%)	19 (33.9%)
**Prior CABG** (%)	7 (12.5%)
**Stroke** (%)	15 (26.8%)
**PAD** (%)	10 (17.9%)
**CHF** (%)	27 (48.2%)
**LVEF** (%) (*n* = 54)	
≥50%	38 (70.4%)
40–49%	8 (14.8%)
<40%	8 (14.8%)
**PHT ^¤^** (%) (*n* = 55)	26 (46.4%)
**AF/AFL** (%)	23 (41.1%)
**Anemia** (%)	53 (94.6%)
**Malignancy** ^†^ (%)	8 (14.3%)

^†^ Active; SD = standard deviation; BMI = Body Mass Index; HTN = hypertension; DLP = dyslipidemia; DM = diabetes mellitus; CAD = coronary artery disease; PCI = percutaneous coronary intervention; CABG = coronary artery bypass graft; PAD = peripheral artery disease; CHF = chronic heart failure; LVEF = left ventricular ejection fraction; PHT = pulmonary hypertension; AF = atrial fibrillation; AFL = atrial flutter. ^¤^ PHT was defined as moderate or greater elevation in pulmonary pressure based on the latest echocardiogram prior to sample collection.

**Table 2:** Dialysis characteristics.

	Total
	N = 56
**Cause of ESRD** ^†^ (%)	
DM	22 (39.3%)
HTN	9 (16.1%)
IgA nephropathy	2 (3.6%)
FSGS	3 (5.4%)
CRS	4 (7.1%)
Amyloidosis	1 (1.8%)
Vasculitis	4 (7.1%)
PKD	2 (3.6%)
GN	2 (3.6%)
Lithium	2 (3.6%)
MM	1 (1.8%)
Uknown	4 (7.1%)
**Dialysis vintage** (SD), months	54.1 (±63.2)
**Dialysis session duration** (SD), hours	3.65 (±0.409)
**Type of solution** (%)	
Standard	41 (73.2%)
Low Ca	10 (17.9%)
High Ca	1 (1.8%)
Standard + KCl	3 (5.4%)
Low Ca + KCl	1 (1.8%)
**Dry weight** (SD), kg	73.8 (±16.4)
**Intradialytic weight gain** (SD), kg	1.42 (±2.72)
**Target loss** (SD), L	2.63 (±0.980)
**MAP pre-dialysis** (SD), mmHg	91.1 (±16.0)
**MAP post-dialysis** (SD), mmHg	84.7 (±13.2)
**Delta MAP ^¤^** (SD), mmHg	6.05 (±16.5)
**Kt/V** (SD)	1.38 (±0.253)

^†^ Presumed etiology of ESRD without pathological confirmation. ^¤^ MAP pre-dialysis minus post-dialysis. ESRD = end-stage renal disease; IgA = immune globulin A; FSGS = focal segmental glomerulosclerosis; CRS = cardio–renal syndrome; PKD = polycystic kidney disease; GN = glomerulonephritis; MM = multiple myeloma; SD =Standard deviation; Ca = calcium; KCl = potassium chloride; kg = kilograms; L = liters; MAP = mean arterial pressure; Kt/V = a measurement of the efficacy of a hemodialysis session.

**Table 3:** Baseline and dialysis characteristics for above/below-median hs-cTnI pre-dialysis.

	Above Median (N = 28)	Below Median (N = 28)	*p*-Value
**Age** (SD), years	74.9 (±12.9)	68.7 (±12.3)	**0.034**
**PHT** (%) (*n* = 55)	17 (60.7%)	9 (32.1%)	**0.076**
**Creatinine pre-dialysis** (SD), mg/dL	6.52 (±1.56)	7.87 (±2.27)	**0.019**
**Creatinine post-dialysis** (SD), mg/dL	2.30 (±0.771)	2.88 (±0.936)	**0.011**
**Cause of ESRD** ^†^ (%)			
DM	11 (39.3%)	11 (39.3%)	**0.013**
HTN	9 (32.1%)	0 (0%)	
IGA nephropathy	1 (3.6%)	1 (3.6%)	
FSGS	0 (0%)	3 (10.7%)	
CRS	2 (7.1%)	2 (7.1%)	
Amyloidosis	1 (3.6%)	0 (0%)	
Vasculitis	0 (0%)	4 (14.3%)	
PKD	0 (0%)	2 (7.1%)	
GN	0 (0%)	2 (7.1%)	
Lithium	2 (7.1%)	0 (0%)	
MM	0 (0%)	1 (3.6%)	
Uknown	2 (7.1%)	2 (7.1%)	
**Intradialytic weight gain** (SD), kg	1.38 (±0.932)	1.47 (±3.77)	**0.03**
**Target loss** (SD), L	2.43 (±0.920)	2.83 (±1.01)	**0.047**
**Number of hospitalizations** (SD)	1.11 (±1.45)	0.536 (±0.999)	**0.0746**

PHT = pulmonary hypertension; ESRD = end-stage renal disease; IgA = immune globulin A; FSGS = focal segmental glomerulosclerosis; CRS = cardio–renal syndrome; PKD = polycystic kidney disease; GN = glomerulonephritis; MM = multiple myeloma; Ca = calcium; KCl = potassium chloride; kg = kilograms; L = liters; Kt/V = a measurement of the efficacy of a hemodialysis session. ^†^ Presumed etiology of ESRD without pathological confirmation.

## Data Availability

All data generated or analyzed during this study are included in this article (and its Supplementary Materials file). The data supporting the findings of this study are not publicly available, as the Institutional Helsinki Committee restricts the release of information beyond the hospital. However, under exceptional circumstances, the committee may evaluate individual requests on a case-by-case basis. For information requests, you may contact the principal investigators, Prof. Asher and Dr. Rabi.

## Supplementary Materials

The following supporting information can be downloaded at https://www.mdpi.com/article/10.3390/jcm14155470/s1, Figure S1: CONSORT 2025 flow diagram; Table S1: Baseline and dialysis characteristics by delta hs-cTnI.
