# Supplementary material for: High Sensitive Cardiac Troponin-I (Hs-cTnI) Levels in Asymptomatic Hemodialysis Patients
Source: J Clin Med. 2025 Aug 4;14(15):5470. doi: 10.3390/jcm14155470 (PMC12347199; doi:10.3390/jcm14155470)
Supplement: Supplementary file 1 [file jcm-14-05470-s001.zip › Supplementary-Table S1.pdf]

Table S1: Baseline and Dialysis characteristics by delta hs-cTnI

	positive† (N=10)	no change (N=17)	negative (N=29)	P-value
**Age** (SD)**,** years	71.8 (16.6)	69.1 (14.9)	73.4 (10.2)	0.564
**BMI** (SD)	29.2 (6.01)	27.8 (5.08)	27.2 (5.22)	0.608
**Male** (%)	6 (60.0%)	11 (64.7%)	18 (62.1%)	0.968
**HTN** (%)	10 (100%)	15 (88.2%)	26 (89.7%)	0.543
**DLP** (%)	8 (80.0%)	14 (82.4%)	25 (86.2%)	0.879
**DM** (%)	8 (80.0%)	12 (70.6%)	15 (51.7%)	0.2
**Smoker** (%) (n=55)	3 (30.0%)	5 (29.4%)	5 (17.2%)	0.76
**Prior CAD** (%)	7 (70.0%)	6 (35.3%)	12 (41.4%)	0.189
**Prior PCI** (%)	5 (50.0%)	4 (23.5%)	10 (34.5%)	0.248
**Prior CABG** (%)	1 (10.0%)	1 (5.9%)	5 (17.2%)	0.513
**PAD** (%)	2 (20.0%)	3 (17.6%)	5 (17.2%)	1
**CHF** (%)	4 (40.0%)	8 (47.1%)	15 (51.7%)	0.81
**PHT** (%) (n=55)	6 (60.0%)	7 (41.2%)	13 (44.8%)	0.181
**AF** (%)	2 (20.0%)	8 (47.1%)	13 (44.8%)	0.324
**Anemia** (%)	9 (90.0%)	16 (94.1%)	28 (96.6%)	0.725
**Creatinine post-dialysis** (SD)**,** mg/dL (n=55)	54.4 (16.7)	62.5 (17.3)	62.2 (19.2)	0.468
**BUN pre-dialysis** (SD), mg/dL (n=55)	54.4 (16.7)	62.5 (17.3)	62.2 (19.2)	0.468
**BUN post-dialysis** (SD), mg/dL	19.1 (8.24)	18.4 (7.01)	19.7 (7.72)	0.866
**WBC** (SD), 10^3/uL	7.58 (2.06)	6.71 (2.17)	7.18 (1.72)	0.504
**Hb** (SD), g/dL	10.9 (1.01)	16.5 (20.0)	10.6 (1.43)	0.202
**MPV** (SD), fL	11.3 (1.32)	11.1 (1.16)	11.1 (1.21)	0.853
**Cause of ESRD**† (%)				
DM	6 (60.0%)	7 (41.2%)	9 (31.0%)	0.314
HTN	1 (10.0%)	0 (0%)	8 (27.6%)	

IGA nephropathy	1 (10.0%)	0 (0%)	1 (3.4%)	
FSGS	0 (0%)	2 (11.8%)	1 (3.4%)	
CRS	0 (0%)	2 (11.8%)	2 (6.9%)	
Amyloidosis	0 (0%)	0 (0%)	1 (3.4%)	
Vasculitis	0 (0%)	1 (5.9%)	3 (10.3%)	
PKD	0 (0%)	2 (11.8%)	0 (0%)	
GN	0 (0%)	1 (5.9%)	1 (3.4%)	
Lithium	0 (0%)	1 (5.9%)	1 (3.4%)	
MM	1 (10.0%)	0 (0%)	0 (0%)	
Uknown	1 (10.0%)	1 (5.9%)	2 (6.9%)	
**Dry weight** (SD), kg	81.0 (19.3)	75.4 (17.0)	70.4 (14.6)	0.194
**Intradialytic weight gain** (SD), kg	2.23 (1.16)	2.05 (1.14)	0.776 (3.53)	0.183
**MAP pre-dialysis** (SD), mmHg	93.0 (15.4)	84.5 (15.7)	94.5 (15.7)	0.229
**MAP post-dialysis** (SD), mmHg	84.1 (15.0)	81.4 (11.5)	86.8 (13.6)	0.612
**Delta MAP**¤ (SD), mmHg	6.44 (21.4)	3.14 (10.3)	7.69 (18.2)	0.85
**KT/V** (SD)	1.35 (0.260)	1.47 (0.267)	1.33 (0.236)	0.202

† Positive delta means increase in hs-cTnI level and vice versa.

¤ MAP pre-dialysis minus post-dialysis

SD =Standard deviation; BMI = Body Mass Index; HTN = Hypertension; DLP = Dyslipidemia; DM = Diabetes Mellitus; CAD = coronary artery disease; CABG = Coronary Artery Bypass Graft; PAD = Peripheral Artery Disease; CHF = Chronic Heart Failure; LVEF = Left Ventricular Ejection Fraction; PHT = Pulmonary Hypertension; AF = Atrial Fibrillation; AFL = Atrial Flutter; ESRD = End Stage Renal Disease; IgA = Immune globulin A; FSGS = Focal Segmental Glomerulosclerosis; CRS = Cardio-Renal syndrome; PKD = Polycystic Kidney Disease; GN = Glomerulonephritis; MM = Multiple Myeloma; Ca = Calcium; KCl = Potassium chloride; Kg = Kilograms; L = Liters; MAP = Mean arterial pressure; Kt/V = a measurement of the efficacy of a hemodialysis session.
