# Supplementary figures and images for: High Sensitive Cardiac Troponin-I (Hs-cTnI) Levels in Asymptomatic Hemodialysis Patients
Source: J Clin Med. 2025 Aug 4;14(15):5470. doi: 10.3390/jcm14155470 (PMC12347199; doi:10.3390/jcm14155470)

## Slide 1
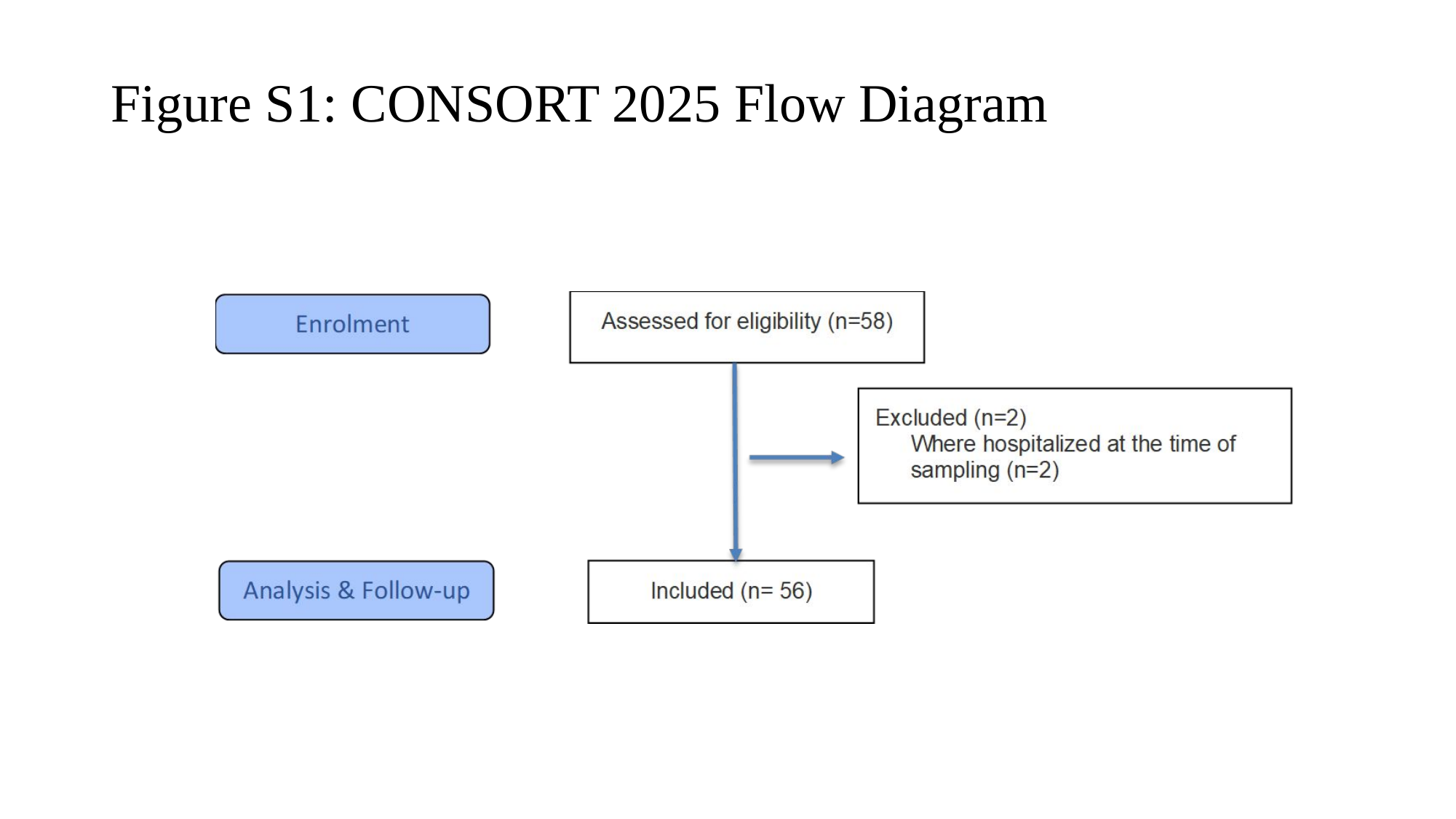

# Figure S1: CONSORT 2025 Flow Diagram

Supplement: Supplementary file 1 [file jcm-14-05470-s001.zip › Figure S1.pptx]
